# Circulating Small Extracellular Vesicles May Contribute to Vaso-Occlusive Crises in Sickle Cell Disease

**DOI:** 10.3390/jcm11030816

**Published:** 2022-02-03

**Authors:** Joanna Gemel, Jared Zhang, Yifan Mao, Gabrielle Lapping-Carr, Eric C. Beyer

**Affiliations:** Department of Pediatrics, University of Chicago, Chicago, IL 60637, USA; jgemel@peds.bsd.uchicago.edu (J.G.); jaredzhang@uchicago.edu (J.Z.); ymao9898@gmail.com (Y.M.); glappingcarr@peds.bsd.uchicago.edu (G.L.-C.)

**Keywords:** exosome, sickle cell anemia, endothelium, vaso-occlusion, acute chest syndrome

## Abstract

We previously found that the plasma of patients with sickle cell disease (SCD) contains large numbers of small extracellular vesicles (EVs) and that the EVs disrupt the integrity of endothelial cell monolayers (especially if obtained during episodes of acute chest syndrome, ACS). The present study was designed to test the generality of this finding to other complications of SCD, specifically to evaluate the possibility that circulating EVs isolated during a vaso-occlusive crises (VOC) also cause damage to the intercellular connections between endothelial cells. Plasma was obtained from nine pediatric subjects at baseline and during VOC episodes. EVs isolated from these samples were added to cultures of microvascular endothelial cells. Immunofluorescence microscopy was employed to assess monolayer integrity and to localize two intercellular junction proteins (VE-cadherin and connexin43). The EVs isolated during VOC caused significantly greater monolayer disruption than those isolated at baseline. The extent of disruption varied between different episodes of VOC or ACS in the same patient. The VOC EVs disrupted the integrity of both junction proteins at appositional membranes. These results suggest that circulating EVs may be involved in modulating endothelial integrity contributing to the pathogenesis of different complications of SCD.

## 1. Introduction

In addition to plasma proteins and blood cells, the blood contains extracellular vesicles (EVs). EVs are produced by many kinds of cells. They contain cellular contents, surrounded by lipid bilayers. EVs have been divided into three groups based on their sizes and processes of formation. Medium and large EVs are generated through cellular damage, while small EVs are actively secreted [1,2,3]. Large EVs (800 nm–5 μm) are produced by plasma membrane disintegration during apoptosis. Microparticles (100–1000 nm) are formed by pinching off from the cell membrane. They are abundantly formed from red blood cells in hemolytic anemias and other blood disorders [4,5,6]. Our studies have focused primarily on the small EVs (50–200 nm) that are often called exosomes. Exosomes are generated by release from the endosomal sorting complex required for transport (ESCRT) [2,7,8]. These small EVs contain various proteins related to the ESCRT (Alix, TSG101, HSC70, and HSP90β). They also contain tetraspanins (including CD63, CD9, and CD81) and nucleic acids (including DNA, mRNAs, and miRNAs) [9]. Following cellular release, EVs from many sources eventually enter the bloodstream. 

Circulating EVs may alter the behavior of the endothelial cells they encounter. This influence may be mediated through direct cell surface interactions or by the transfer of contents (such as proteins or nucleic acids), allowing the passage of signals from the cell of origin. Plasma EVs can modulate pathophysiologic components of cardiovascular diseases such as inflammation, endothelial dysfunction and damage, thrombosis, and ischemia-reperfusion injury [10,11]. The abundance of circulating EVs (microvesicles and exosomes) is increased in patients with different cardiovascular diseases (reviewed by [10,12]). These EVs derive from white blood cells, red blood cells, platelets, and endothelial cells. Several studies have shown that EVs derived from blood cells alter the adhesiveness of endothelial cells, cause them to assume a more inflammatory phenotype, and/or change expression of various cytokines and adhesion proteins [13,14,15,16,17]. Measurements of EVs and/or their contents can be useful as biomarker assessments of disease [12,18,19,20].

We have been focusing on the roles of EVs in the pathophysiology of sickle cell disease (SCD). Although SCD is ultimately due to a single amino acid substitution in one of the hemoglobin polypeptides (Glu6→Val in β-globin), endothelial activation and damage are central to many of the complications of this disease [21]. Polymerization of the abnormal hemoglobin can deform erythrocytes. The rigidity and abnormal shapes of sickled erythrocytes cause intermittent occlusion of small blood vessels. Repetitive ischemic insults and ischemia/reperfusion injuries may cause damage to many different organs. The locations and extent of damage differ between individuals with SCD, but common sites include the bones, lungs, spleen, brain, heart, skin, and kidneys. Endothelial injury and activation of an inflammatory phenotype also contribute to the vaso-occlusion through changes such as increased expression or exposure of various adhesion molecules [22,23]. The presence of increased levels of medium-sized and small EVs in subjects with SCD suggests that they might also be involved in some of the endothelial alterations in SCD (reviewed by [6,24]). Some of these EVs have been implicated in accentuating pro-thrombotic or inflammatory changes in SCD [25,26,27,28,29].

Our recent studies have implicated small EVs in the pathogenesis of SCD complications. We found that small EVs are abundantly present within the plasma of children and young adults with SCD [30,31]. These EVs have the size expected for exosomes (~100 nm diameter), and they contain typical exosomal proteins (CD63 and flotillin-1) [32]. Several different kinds of techniques have demonstrated that the SCD EVs cause disruption of intact monolayers of cultured endothelial cells: impedance is reduced, spaces open between cells, and adherens junctions containing VE-cadherin are reduced in abundance or disrupted [30,31,32]. The SCD EVs also cause disruption of other classes of intercellular junctions containing ZO-1 or connexin43 [31,33]. The endothelial disruption caused by EVs is more severe if the EVs are obtained during an episode of acute chest syndrome (ACS) compared to baseline samples obtained from the same subject [32].

We sought to examine the generality of the effects of EVs in contributing to different vascular consequences of SCD and the specificity of attributing the effects to the EVs. In our prior studies, we had only examined the effects of EVs obtained from children with SCD obtained at baseline or during episodes of one disease complication, ACS. The most common complications of SCD are vaso-occlusive pain crises (VOC) due to painful occlusions of vessels in the bone. The current study examined the effects of EVs isolated from subjects during a severe VOC episode as compared to baseline samples or samples obtained during an ACS episode. We also utilized a different EV isolation procedure allowing increased confidence that results can be attributed to the EVs.

## 2. Materials and Methods

### 2.1. Subjects and Event Characteristics 

The sickle cell disease registry and biobank at the University of Chicago prospectively enrolled subjects seen at Comer Children’s Hospital and La Rabida Children’s Hospital. Parents or patients greater than 18 years of age provided informed consent. Assent was also obtained from 9–18-year-olds. Protocols were approved by the University of Chicago Institutional Review Board (protocol # 14-0466 and 15-0263). All studies were conducted in accordance with the guidelines set by the Declaration of Helsinki.

We queried the biobank to find subjects who had baseline plasma samples as well as samples obtained at the beginning of an admission for VOC or ACS. We identified nine such patients. Baseline (steady state) samples were obtained from subjects with SCD at the time of a “well” clinic visit when they had no infections or significant pain. Subjects had not received blood transfusions for at least 4 weeks prior to obtaining this baseline blood sample. Control subjects (*n* = 3) were recruited from patients who were being seen in the general pediatric and benign hematology clinics and were having blood drawn for screening purposes or testing for iron deficiency. None of the control subjects had obesity, asthma, or another inflammatory disorder.

All episodes of VOC or ACS were of substantial severity requiring hospital admission. Vaso-occlusive crises were defined as acute episodes of pain requiring treatment with parenteral opioids that were not accompanied by other signs or symptoms (such as fever) suggesting another etiology. ACS episodes were defined as the appearance of a new infiltrate on chest X-ray accompanied by fever, requirement for supplemental oxygen, tachypnea, cough, or chest pain. None of the patients required transfer to the intensive care unit or exchange transfusion for the episodes included in this study. Clinical and demographic characteristics of the subjects with SCD are shown in Table 1. Asthma was defined either by electronic medical record documentation of an ICD code for asthma or by prescription of a long-acting controller asthma medication. Splenectomy and cholecystectomy were identified by procedure codes. Obstructive sleep apnea was diagnosed by sleep study.

### 2.2. Blood Collection and EV Isolation

Blood samples for SCD subjects at baseline and for control subjects were obtained during an outpatient clinic appointment. Blood samples for SCD subjects experiencing episodes of VOC or ACS were obtained during the first 24 h of a hospital admission. Professional phlebotomists collected the blood (~6 mL) into EDTA-containing (lavender top) tubes. The blood was centrifuged at 2500× *g* at 24 °C for 15 min; platelet-free plasma was transferred to a new tube without disturbing the buffy coat or pellet, and the sample was re-centrifuged under identical conditions. Supernatants were frozen in aliquots and stored at −80 °C. Thawed aliquots of samples were centrifuged at 1500× *g* for 10 min at 24 °C; then, the supernatant was transferred to a new tube and centrifuged at 10,000× *g* for 10 min at 24 °C prior to isolation of EVs [32].

EVs were isolated by size exclusion, using qEV-35nm single columns (Izon Science, Medford, MA, USA) following the manufacturer’s instructions. A total of 230 µL of plasma was used as starting material for isolations. Samples were centrifuged at 1500× *g* for 10 min to remove any cells and large particles, then the supernatant was gently moved to a new tube and centrifuged again at 10,000× *g* for 10 min. To avoid contamination with any pellet, only 200 µL of supernatant was removed and used for the column purification. A total of 200 µL fractions were collected. Immunoblotting showed that fractions 6 and 7 contained the greatest content of two exosomal proteins (CD63 and flotillin-1). These fractions were combined and concentrated to 50 µL using Microcon 30 K centrifugal filter devices (SIGMA-Aldrich, Saint Louis, MO, USA) for experimental studies [32].

### 2.3. Nanoparticle Tracking Analysis and Transmission Electron Microscopy 

A NanoSight NS300 (Malvern Panalytical Inc., Westborough, MA, USA) was used to perform Nanoparticle tracking analysis. The EV samples were diluted 1:100–1:150 in PBS and then injected into the 488 nm laser chamber. A syringe pump provided constant output. For each sample, at least three recordings were performed. Nanoparticle size was determined using the nanoparticle tracking analysis software. 

Imaging of EVs negatively stained with uranium acetate was performed by transmission electron microscopy in the Advanced Electron Microscopy Core Facility at the University of Chicago with the assistance of Dr. Tera Lavoie. An FEI Tecnai G2 300 kV Super Twin Electron Microscope, (FEI Company, Hillsboro, OR, USA) was used.

### 2.4. Primary Endothelial Cell Culture

Human dermal microvascular endothelial cells (CC-2543, HMVEC-D) were purchased from Lonza (Allendale, NJ, USA). The cells were cultured in endothelial growth medium (EGM-2MV Bullet Kit; Lonza) at 37 °C in an atmosphere containing 5% CO_2_. All experiments were performed at passage 10.

### 2.5. Antibodies 

VE-cadherin was detected using a mouse monoclonal antibody (sc-9989, Santa Cruz Biotechnology Inc., Dallas, TX, USA) at 1:100 dilution for immunofluorescence and at 1:2000 dilution for immunoblotting. Connexin43 (Cx43) was detected using rabbit polyclonal antibodies directed against amino acids 363–382 of human/rat Cx43 (C6219, SIGMA-Aldrich, Saint Louis, MO, USA) at 1:250 dilution for immunofluorescence and at 1:5000 dilution for immunoblotting. Immunoblotting for EV markers was performed using primary mouse monoclonal antibodies anti-flotillin-1 (sc-133153, Santa Cruz, CA, USA) diluted 1:200 and anti-CD63 (sc-5275, Santa Cruz, CA, USA) diluted 1:500. The secondary antibodies, AlexaFluor 488 goat anti-mouse IgG and horseradish peroxidase (HRP)-conjugated goat anti-rabbit or anti-mouse IgG antibodies, were obtained from Jackson ImmunoResearch (West Grove, PA, USA). Immunoblotting was performed as described earlier [32,33].

### 2.6. Immunohistochemistry of Endothelial Cells 

For microscopy studies, endothelial cells were cultured on glass coverslips that had been pre-coated with 5 µg/mL fibronectin and 0.02% gelatin (SIGMA-Aldrich) for 5 min. To study the effects of EVs, confluent monolayers of cells were treated for 48 h by adding fresh growth medium alone or medium containing an appropriate dilution of EVs. Coverslips were prepared for immunofluorescence by fixation in 4% paraformaldehyde, permeabilization with 1% Triton X-100, and blocking using PBS containing 10% normal goat serum and 1% Triton X-100. Immunofluorescent staining for VE-cadherin and for Cx43 were performed as previously described [32,33]. Nuclear counter staining was performed by incubating fixed and permeabilized cells for 15 min with 500 µg/mL DAPI (4′,6-Diamidino-2-Phenylindole Dihydrochloride). After mounting coverslips on glass slides using Prolong Gold anti-fade reagent (Thermo Fisher Scientific Inc., Waltham, MA, USA), the slides were sealed and stored in the dark at 4 °C. Microscopic examination of stained cells was performed using the 40× Plan Apochromat objective in a Zeiss Axioplan 2 microscope, and microphotographs were obtained using an Axiocam digital camera using the Zeiss AxioVision software (Jena, Germany). Lower power micrographs were obtained using the 10× objective.

The team members who performed microphotography and image analysis were unaware of the source of EVs (control vs. baseline vs. VOC vs. ACS) that had been applied to the different coverslips they were analyzing. 

Images were analyzed using the Image J software (http://rsb.info.nih.gov/ij/; accessed on 11 July 2014). Monolayer disruption was quantified by determining the percentage of intercellular space (cell-free area) within each microscopic image field as described earlier [32,33].

### 2.7. Statistical analysis

All data were expressed as the mean ± standard error of the mean. When comparing more than two populations, we performed analysis of variance (ANOVA) with the Tukey post hoc multiple comparison test. *p* < 0.05 was considered to be statistically significant. 

## 3. Results

### 3.1. Subject Characteristics

We identified nine subjects from within our SCD biobank who had blood samples obtained at baseline and at the beginning of a hospitalization for VOC. Three control subjects of similar ages (12, 14, and 15 years) including both males (1) and females (2) were also identified; they all had mild anemia (hemoglobin ~10 gm/dL), but they did not have other abnormal hematologic values. (Only a limited number of control samples were available because they came from children without sickle cell disease. Logistically and ethically, they are hard to obtain and therefore precious. Because we have previously compared the effects of EVs from SCD and control subjects, the control samples were only used to confirm that they were non-toxic in the various experiments and were not used extensively.)

The demographic and clinical characteristics and some hematological laboratory values for the SCD subjects are shown in Table 1. The ages, clinical data, and laboratory values all correspond to the time of baseline blood draw or to the time of the VOC or ACS episodes. Seven subjects had the SS genotype, and two had the SC genotype. Many parameters (including white blood cell, reticulocyte, or platelet counts and mean corpuscular volume, MCV) did not differ significantly between baseline and VOC episodes. The subjects were significantly more anemic when admitted for VOC (smaller hemoglobin values).

### 3.2. Properties of Extracellular Vesicles

We have previously characterized the small EVs that we isolated from control subjects and from subjects with SCD (at baseline or during ACS episodes) using two different isolation methods: precipitation and size-exclusion chromatography [31,32,33]. For the current study, we only used size-exclusion chromatography because it produced EVs with much less plasma protein contamination. The VOC samples had some similar characteristics to the other SCD samples (obtained at baseline or during ACS episodes) that we have studied previously [31,32,33]. Nanoparticle Tracking Analysis showed that the EVs were distributed within a single peak with a mode diameter of 95–100 nm (Supplementary Figure S1). By transmission electron microscopy, the preparations contained relatively homogeneous populations of vesicles (Supplementary Figure S2). However, the particles appeared rather small, likely due to shrinkage during sample processing [34,35]. 

Immunoblots for the presence of proteins found in exosomes, or small EVs, confirmed the presence of both CD63 and flotillin-1 (Supplementary Figure S3). By NTA analysis, we found that the particle concentrations were rather variable, but they did not differ significantly between samples obtained at baseline (3.8 ± 1.0 × 10^10^, *n* = 3) or during VOC (1.8 ± 0.5 × 10^10^, *n* = 3). Moreover, NTA analysis of a single control sample gave a similar particle concentration (4.0 × 10^10^).

**Figure 1:**
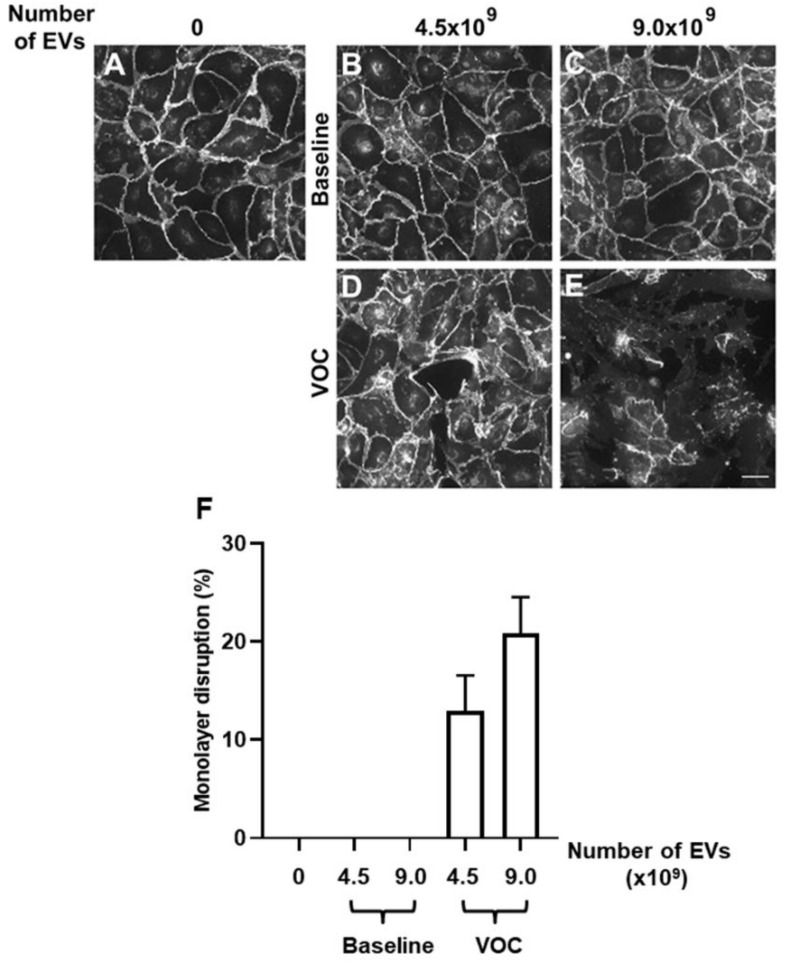
Dose-dependent endothelial monolayer disruption by EVs from a subject with SCD (at baseline or during VOC). Human endothelial cell monolayers were treated with no EVs or with 4.5 × 10^9^ or 9.0 × 10^9^ EVs (in 250 µL of tissue culture medium). After 48 h, cells were fixed, and VE-cadherin was detected by immunofluorescence. Representative photomicrographs are shown for cell monolayers following treatment with no EVs (**A**), or following treatment with 4.5 × 10^9^ (**B**,**D**) or 9.0 × 10^9^ (**C**,**E**) EVs isolated from a subject with SCD at baseline (**B**,**C**) or at the beginning of an episode of VOC (**D**,**E**). The percentage of monolayer disruption was calculated as described in Materials and Methods (based on pictures taken from at least 6 fields per sample). The graph (**F**) shows the average percentage of disruption (±SEM). No disruption was detected in cells treated with no EVs or cells treated with either dose of EVs from the baseline samples; in contrast, disruption was seen with EVs from the VOC samples and increased between the two doses (*p* < 0.005 for either VOC sample vs. no EVs or baseline, and *p* < 0.005 between VOC doses). For the examples shown, the monolayer disruption was 4.8% in panel **D** and 21.7% in panel E.

Previously, we studied how plasma EVs from subjects with SCD (at baseline or during an episode of ACS) affected the integrity of endothelial cell monolayers, and we quantified the extent of damage in immunofluorescence micrographs [32]. Our prior studies showed that there was little effect of the EVs from subjects with SCD until 24–48 h after application to the cells. In the current study, we screened multiple doses of EVs for their effects on endothelial monolayer integrity. We identified two doses (4.5×10^9^ and 9.0×10^9^) at which monolayers treated with EVs obtained during a VOC episode caused substantial disruption, and the extent of disruption increased between doses (from 13% to 21% in the experiments shown in Figure 1). At these doses, monolayers treated with EVs obtained at baseline caused no detectable disruption (Figure 1); these monolayers looked similar to those treated with no EVs. Based on these results, we used the lower dose for all samples in subsequent experiments. 

**Figure 2:**
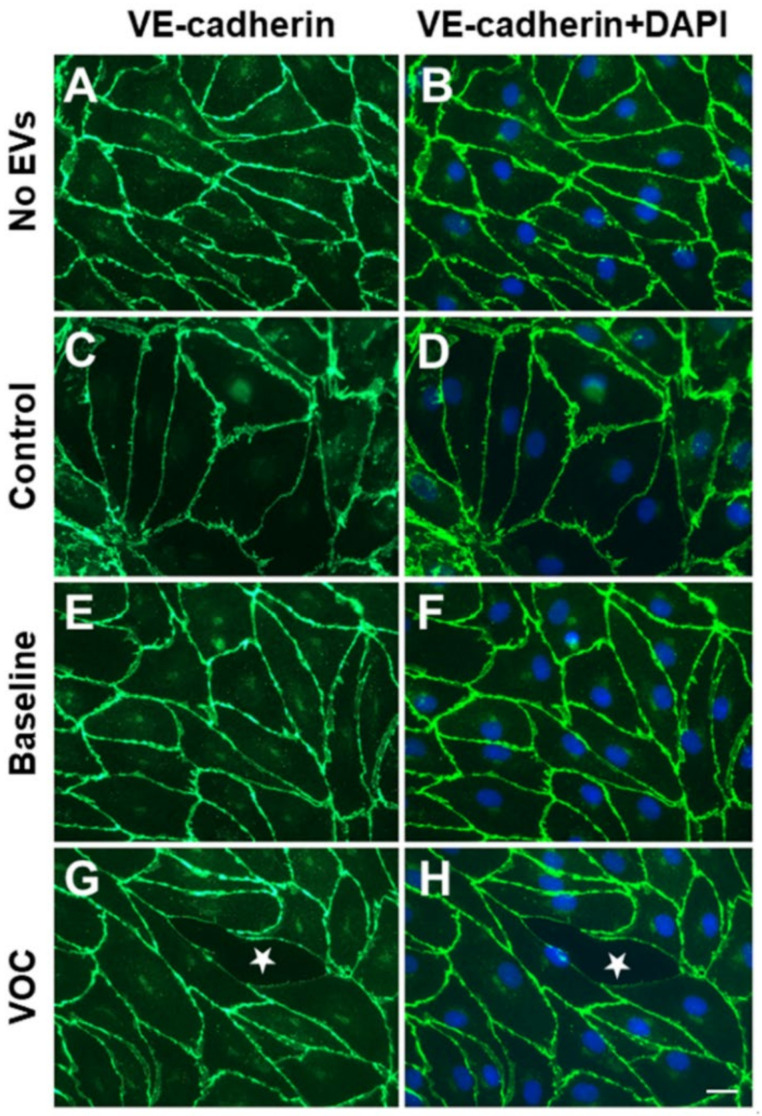
Disruption of endothelial monolayer integrity by EVs is shown in representative photomicrographs of cells obtained 48 h after treatment with no EVs (**A**,**B**), EVs from a control subject (**C**,**D**), EVs from a subject with SCD at baseline (**E**,**F**), and EVs from the same subject at the beginning of an episode of VOC (**G**,**H**). VE-cadherin was detected by immunofluorescence (green) and nuclei were detected by staining with DAPI (blue). In the example shown in the bottom row for a VOC sample, the monolayer disruption was 5.6%. The white star indicates unoccupied space that has opened between cells. Scale bar represents 20 µm.

We examined changes in the appearance of confluent endothelial monolayers following treatment with EVs using fluorescence microscopy. Representative examples of these photomicrographs are shown in Figure 2 and Supplementary Figure S4. Cultures were treated with no EVs, with EVs from controls, or with EVs from subjects with SCD at baseline or during VOC. VE-cadherin was localized by immunofluorescence (Figure 2A,C,E,G) and nuclei were detected by staining with DAPI (Figure 2B,D,F,H). The cellular appearance was similar for cultures treated with no EVs or with EVs from control subjects (Figure 2A–D and Supplementary Figure S4). VE-cadherin was abundant in a continuous distribution along cell membranes at points of contact between cells. Few or no spaces separating cells were observed. Under the conditions used for these experiments, endothelial cells treated with EVs isolated from subjects with SCD obtained at baseline did not differ in appearance from the cells treated with no EVs or control EVs; they did not show spaces between cells (Figure 2E,F). In contrast, cells treated with EVs obtained during an episode of VOC looked markedly different (Figure 2G,H and Supplementary Figure S4). Many of the endothelial cells were separated by cell-free spaces; VE-cadherin localization was thick, convoluted, and had frequent discontinuities. DAPI staining of nuclei appeared similar in all cells, regardless of treatments.

**Figure 3:**
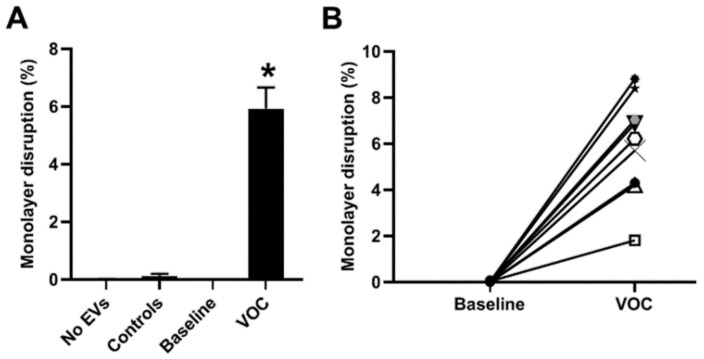
Quantitation of micrographs shows that EVs isolated during VOC disrupt endothelial monolayers in vitro. Cultures of endothelial cells were treated with EVs for 48 h, processed for immunofluorescence microscopy, and then analyzed to calculate the percentage of monolayer disruption. (**A**) This graph shows the average percentage of monolayer disruption (±SEM) for cells treated with no EVs, EVs from control subjects (*n* = 3), EVs from 9 subjects with SCD obtained at baseline, and EVs obtained from the same 9 subjects at the beginning of VOC episodes. There were no significant differences among no EVs, EVs from control subjects, and baseline EVs isolated from SCD patients. However, EVs isolated from SCD subjects during VOC episodes caused significantly more disruption than those in any of the other groups (*, *p* < 0.0001). (**B**) This graph shows the percent disruption in individual patients at baseline or during a VOC episode. Each patient is indicated by a different symbol, and results from the same patient are connected by lines. All of the SCD patients showed an increase in monolayer disruption between baseline and VOC.

We performed multiple experiments using the EVs prepared from all nine subjects with SCD (isolated both at baseline and during a VOC episode) and took photomicrographs similar to those shown in Figure 2. We quantified the extent of damage to the endothelial monolayers by calculating the area of intracellular space as a percentage of the total image area (as described in [32]). Endothelial cells treated with no EVs, with EVs from control subjects, or with EVs collected from subjects with SCD at baseline showed few or no open spaces within the monolayers (Figure 3A). EVs isolated from the same subjects during episodes of VOCs caused significant disruption of the endothelial monolayer (on average ~6% for the samples shown in Figure 3A). 

We also compared the extent of monolayer disruption caused by samples from the same subject at baseline or during VOC (Figure 3B). EVs isolated from all subjects caused increased disruption during VOC. Endothelial disruption did not differ based on genotype. The extent of disruption increased from baseline to VOC in both SC subjects (from 0 to 6.9% and from 0 to 5.7%). The average amount of disruption was 5.8% for EVs obtained during VOC episodes from the SS subjects.

### 3.3. Endothelial Monolayer Disruption Varies with Different Episodes of VOC or ACS 

**Figure 4:**
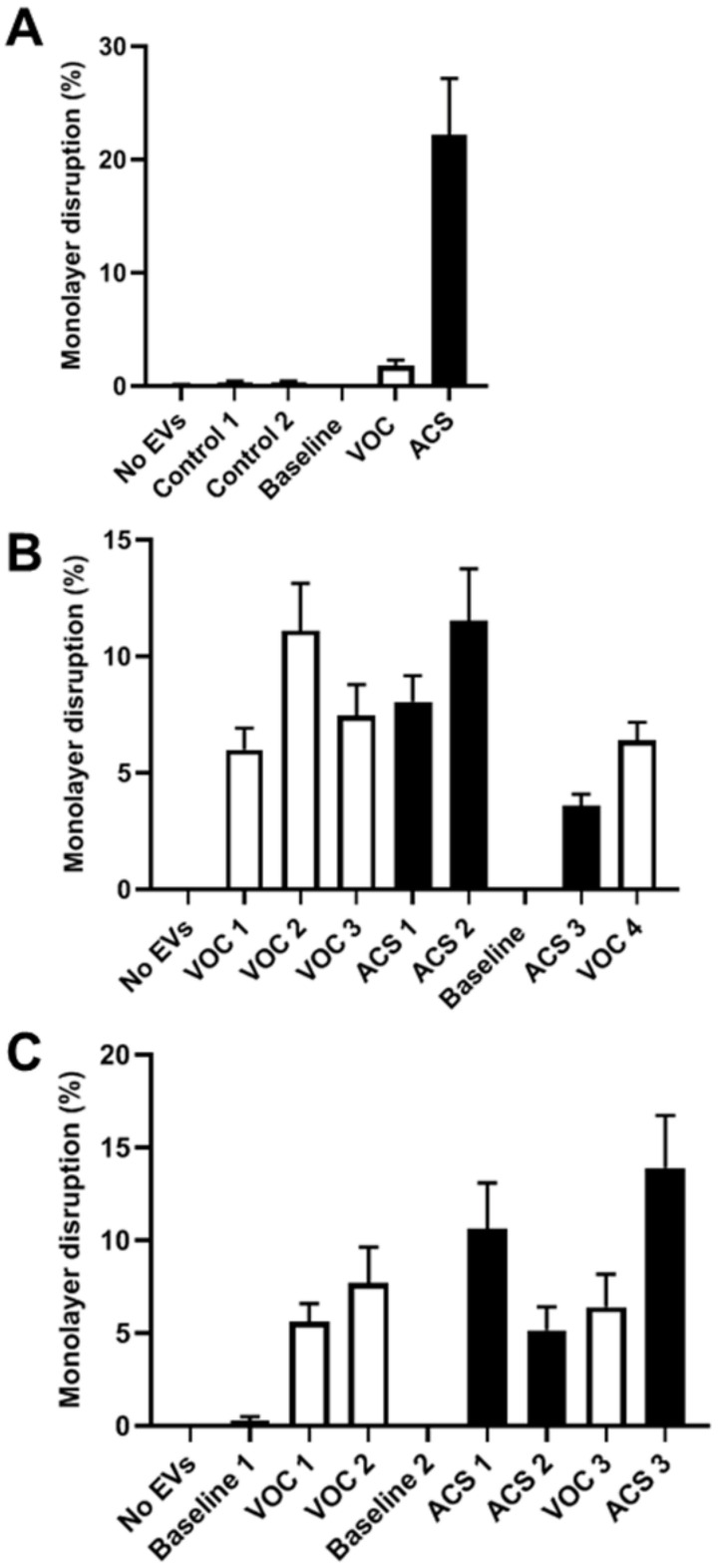
Comparison of the endothelial monolayer disruption at different time points by EVs isolated at baseline or during VOC or ACS episodes in three patients. Three different patients (**A**–**C**) were studied. Episodes of VOC and ACS (and baseline samples) are shown in chronological order. Cultures of endothelial cells were treated with EVs for 48 h, processed for immunofluorescence microscopy, and then analyzed to calculate the percentage of monolayer disruption. Graphs show the average percentage of disruption (± SEM). Each experiment also included no EVs and/or EVs from control subjects. All samples were studied at the same time using the same endothelial cultures, and they were photographed using identical settings. Subject A was a female who was 6 years and 8 months old when the initial baseline sample was obtained; subsequent samples were obtained at hospitalizations over the next 2.5 years. Subject B was a male who was 6 years and 0 months old at the time of the first VOC episode; subsequent samples were obtained at hospitalizations over the next 2 years and 9 months. Subject C was a female who was 4 years and 1 month old at the time of the baseline sample; subsequent samples were obtained at hospitalizations over the next 3 years.

We also wanted to compare the monolayer disruption caused by EVs obtained during episodes of VOC and those obtained during episodes of ACS and between different episodes of VOC or ACS in the same patient. Three of our subjects had samples obtained during both episodes of VOC and of ACS (Figure 4). The clinical characteristics and hematologic values for this subset of patients are shown in Table 1. All three subjects had baseline samples obtained before either VOC or ACS episodes, and two subjects (B and C) had additional baseline samples obtained during relatively healthy periods between VOC or ACS episodes. (The early baseline sample for subject B is not shown because it was not analyzed simultaneously with the other samples from this subject. In a separate experiment, it caused no disruption.) None of the baseline samples produced much disruption of the endothelial monolayers. All VOC and ACS samples caused significantly more disruption than no EVs or baseline EVs. However, the extent of disruption was quite variable between episodes. While many of the ACS and VOC episodes did not differ from each other, a few (subject A, ACS; subject B, VOC2 and ACS2; subject C, ACS3) caused more disruption than other episodes in the same patient.

### 3.4. EVs Isolated from Subjects with VOC also Disrupt Endothelial Gap Junctions

**Figure 5:**
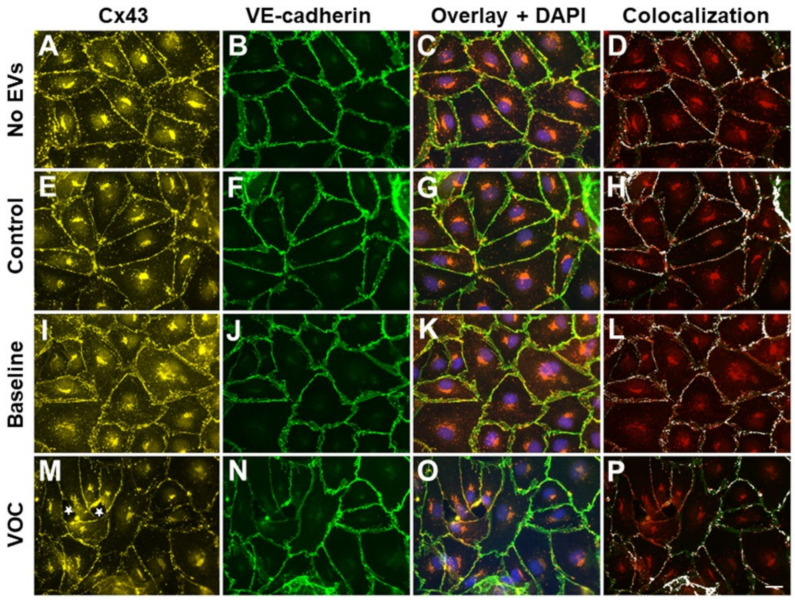
EVs obtained during VOC crises disrupt different classes of endothelial intercellular junctions. Representative photomicrographs are shown for human endothelial cell monolayers following 48 h treatment with no EVs (**A**–**D**), EVs from a control subject (**E**–**H**), EVs from a subject with SCD at baseline (**I**–**L**), and EVs from the same subject at the beginning of an episode of VOC (**M**–**P**). Cx43 (**A**,**E**,**I**,**M**) and VE-cadherin (**B**,**F**,**J**,**N**) were detected by immunofluorescence. The third column of panels (**C**,**G**,**K**,**O**) shows an overlay of the Cx43 and VE-cadherin images and shows the localization of nuclei stained with DAPI (blue). The right panels (**D**,**H**,**L**,**P**) are also overlap images of the Cx43 and VE-cadherin immunofluorescence in which co-localization between Cx43 and VE-cadherin is shown in white. The extent of co-localization at the membrane decreased in the cells treated with EVs obtained during the VOC episode. White stars indicate spaces between cells. In the example shown in the bottom row for the VOC sample, the monolayer disruption was 1.6%. Scale bar represents 20 µm.

We have previously found that small EVs from patients with VOC also disrupted other kinds of intercellular junctions as well as the adherens junctions containing VE-cadherin [33]. Therefore, we tested the effect of EVs from a SCD patient with an ACS episode on the abundance and distribution of the gap junction protein Connexin43 (Cx43) (Figure 5). We found few differences in the distribution of Cx43 (or its overlap with VE-cadherin) in endothelial monolayers treated with no EVs (Figure 5A–D), control EVs (Figure 5E–H), or baseline EVs (Figure 5I–L). However, treatment with EVs obtained during an episode of VOC (which caused the opening of some spaces in the monolayer) caused the abundance of Cx43 to decrease substantially between some cells, to become less continuous, and to show less overlap with VE-cadherin (Figure 5M–P). We also performed immunoblots to characterize the levels of Cx43 and of VE-cadherin in endothelial cell cultures treated with EVs. As shown in Supplementary Figure S5, we found that EVs isolated from a subject with VOC caused a decrease in levels of both Cx43 and VE-cadherin as compared to EVs obtained from the same subject at baseline.

## 4. Discussion

The importance of different kinds of EVs in the pathogenesis of SCD is increasingly becoming appreciated (as reviewed by [36]). Several different roles have been documented for medium-sized EVs (microparticles) derived from red cells, monocytes, platelets, and endothelial cells [6].

In this paper, we have presented several experiments that extend our previous studies implicating EVs circulating in the plasma of subjects with SCD in vascular complications of that disease. We previously showed that subjects with SCD contain an increased abundance of EVs in their plasma (compared to control subjects) that have multiple characteristics consistent with exosomes [31,32]. Our past studies using the same endothelial cell culture model system showed that these EVs cause damage to monolayers of cultured endothelial cells: baseline EVs from subjects with a history of ACS cause more damage than those with no such history [31], and EVs isolated during ACS episodes are more toxic than EVs isolated at baseline. Moreover, we have previously shown that the observed effects require intact vesicles since mechanical disruption of the EVs by sonication abolishes monolayer disruption [32]. Our current results show that EVs isolated during VOC episodes are also more toxic than EVs isolated at baseline. As in our previous studies, the current data show that EVs obtained from subjects with SCD during crises (VOC) disrupt multiple classes of endothelial intercellular junctions. Although we did not assess electrical coupling or dye transfer in the current study, a similar extent of Cx43 disruption was sufficient to reduce intercellular communication previously [33].

Our new data extend our previous studies in other ways. The patients in the current study were somewhat older than those in the previous studies, and several had experienced complications of the disease requiring splenectomy or cholecystectomy. The current study includes two subjects that were compound heterozygotes for Hemoglobin S and Hemoglobin C, demonstrating that small EVs may contribute to endothelial damage in these individuals as well as in those with the SS genotype.

Several studies have implicated endothelial barrier disruption in the pathophysiology of sickle cell disease complications such as acute chest syndrome [37,38]. Evidence supports various molecules being released or generated by hemolysis (such as free hemoglobin, heme, and hemin) as possible mediators of this disruption [39,40,41,42]. While the plasma of our patients must contain these agents, we previously showed that the concentrations in our EV preparations were too low to affect our endothelial cells [32]. Therefore, we suggest that small EVs should be added to the list of possible explanations for endothelial barrier disruption in sickle cell disease.

The current results do not allow us to determine if there is a difference in the effects of EVs obtained during ACS or VOC episodes. EVs isolated during both kinds of crises caused much more damage to the endothelial cells (and their intercellular connections) than baseline or control EVs. While every episode caused monolayer disruption, the extent was quite variable between different episodes. We currently cannot explain this variability (even for different episodes in the same subjects). The significant heterogeneity between patients with sickle cell disease and across their lifetime is a well-known phenomenon, but a future study with a large cohort could help answer this question. We have analyzed some clinical data for the patients (hemoglobin, WBC, platelet count, reticulocyte count, bilirubin, that were obtained at the same blood draws as the EVs), but there was no correlation with the extent of monolayer disruption (data not shown). To maximize consistency, all experiments were performed using the same kinds of endothelial cells (dermal microvascular cells). Although ACS and VOC both have components of a microvascular pathogenesis, they are clearly clinically distinct events and must reflect differences in lung vs. bone vasculature. In the future, it may be interesting to repeat some of these experiments using endothelial cells that more closely model those organ sites.

The endothelial monolayer disruption caused by SCD EVs is a complex cellular process that we have previously modeled [24] involving a time-dependent disruption of different classes of intercellular junctions connecting endothelial cells [32,33]. The data presented in the current manuscript confirm that VOC EVs (as ACS EVs) disrupt endothelial cell adherens junctions and gap junctions. Moreover, this disruption requires about 2 days to be substantial. This time course suggests changes in gene expression and cellular remodeling and no immediate pharmacological changes. This suggests that the EVs affect their target cells by modulating gene expression and/or cellular signaling; these events might occur at the cell surface or after delivery of EV cargo into the cell. These are mechanistic issues similar to those of EVs in many different systems. Currently, we have limited information about the contents of our EVs. We have established that the EVs contain microRNAs, as expected (unpublished data). Some of these microRNAs may be responsible for initiating the endothelial cell changes. Our next steps will include comprehensive analysis of the EV contents (including lipids, proteins, and nucleic acids) and changes in endothelial cell gene expression.

## Figures and Tables

**Table 1:** Demographic data, clinical characteristics, and hematologic values of subjects with sickle cell disease.

	Baseline (*n* = 9)	VOC (*n* = 9)	*p*-Value	ACS (*n* = 3)	*p*-Value
**Demographic Data**					
Age in years, median (range)	12 (2–16)	13 (3–16)	0.065	7 (5–9)	ns
Male, n (%)	5 (56)		ns	1 (33)	ns
Female, n (%)	4 (44)			2 (67)	
**Clinical Characteristics** **(*n* (%))**					
Genotype SS SC		7 2		3 0	
Hydroxyurea		6 (67)		1 (33)	ns
Asthma		3 (33)		1 (33)	ns
Obstructive Sleep Apnea		3 (33)		1 (33)	ns
Splenectomy		3 (33)		0	ns
Cholecystectomy		2 (22)		0	ns
**Hematologic Values (median (range))**					
White blood cell count (×10^3^/µL)	9.75 (5.3–18.9)	10.6 (6.4–29.3)	ns	15.7 (13.1–18.5)	ns
Hemoglobin (g/dL)	9.8 (7.2–11.1)	7.7 (6.0–9.8)	0.007	7.6 (6.9–8.0)	0.006
Mean Corpuscular Volume (fL)	84.8 (74.3–99.8)	83.3 (63.5–102.5)	ns	86.0 (73.8–89.6)	ns
Absolute reticulocyte count (×10^3^/µL)	266 (169–543)	248 (163–478)	ns	477 (187–549)	ns
Platelets (×10^3^/µL)	367 (134–595)	252 (115–427)	ns	293 (282–365)	ns

ns = not statistically significant.

## Supplementary Materials

The following are available online at https://www.mdpi.com/article/10.3390/jcm11030816/s1, Figure S1: Representative Nanoparticle Tracking Analysis profile of EVs isolated from platelet-free plasma of an SCD patient. Figure S2: Transmission electron micrograph illustrates the appearance of EVs isolated by size-exclusion chromatography from the platelet-free plasma of a patient with SCD after negative staining. Figure S3: Properties of EVs isolated by size-exclusion chromatography. Figure S4: Representative low magnification photomicrographs of endothelial monolayer integrity and its disruption by EVs. Figure S5: Levels of Cx43 and VE-cadherin are decreased in endothelial cells treated with EVs obtained during a VOC episode.

## References

[1] Colombo M., Raposo G., Théry C. (2014). Biogenesis, secretion, and intercellular interactions of exosomes and other extracellular vesicles. Annu. Rev. Cell Dev. Biol..

[2] Cocucci E., Meldolesi J. (2015). Ectosomes and exosomes: Shedding the confusion between extracellular vesicles. Trends Cell Biol..

[3] Meldolesi J. (2018). Exosomes and ectosomes in intercellular communication. Curr. Biol..

[4] Morel O., Jesel L., Freyssinet J.-M., Toti F. (2011). Cellular mechanisms underlying the formation of circulating microparticles. Arterioscler. Thromb. Vasc. Biol..

[5] Westerman M., Porter J.B. (2016). Red blood cell-derived microparticles: An overview. Blood Cells Mol. Dis..

[6] Hebbel R.P., Key N.S. (2016). Microparticles in sickle cell anaemia: Promise and pitfalls. Br. J. Haematol..

[7] Théry C., Zitvogel L., Amigorena S. (2002). Exosomes: Composition, biogenesis and function. Nat. Rev. Immunol..

[8] Van Niel G., D’Angelo G., Raposo G. (2018). Shedding light on the cell biology of extracellular vesicles. Nat. Rev. Mol. Cell Biol..

[9] Théry C., Witwer K.W., Aikawa E., Alcaraz M.J., Anderson J.D., Andriantsitohaina R., Antoniou A., Arab T., Archer F., Atkin-Smith G.K. (2018). Minimal information for studies of extracellular vesicles 2018 (MISEV2018): A position statement of the International Society for Extracellular Vesicles and update of the MISEV2014 guidelines. J. Extracell. Vesicles.

[10] Ridger V.C., Boulanger C.M., Angelillo-Scherrer A., Badimon L., Blanc-Brude O., Bochaton-Piallat M.-L., Boilard E., Buzas E.I., Caporali A., Dignat-George F. (2017). Microvesicles in vascular homeostasis and diseases. Position Paper of the European Society of Cardiology (ESC) Working Group on Atherosclerosis and Vascular Biology. Thromb. Haemost..

[11] Oggero S., Austin-Williams S., Norling L.V. (2019). The contrasting role of extracellular vesicles in vascular inflammation and tissue repair. Front. Pharmacol..

[12] Jansen F., Nickenig G., Werner N. (2017). Extracellular vesicles in cardiovascular disease: Potential applications in diagnosis, prognosis, and epidemiology. Circ. Res..

[13] Barry O.P., Praticò D., Savani R.C., FitzGerald G.A. (1998). Modulation of monocyte-endothelial cell interactions by platelet microparticles. J. Clin. Investig..

[14] Brodsky S.V., Zhang F., Nasjletti A., Goligorsky M.S. (2004). Endothelium-derived microparticles impair endothelial function in vitro. Am. J. Physiol. Heart Circ. Physiol..

[15] Mezentsev A., Merks R.M.H., O’Riordan E., Chen J., Mendelev N., Goligorsky M.S., Brodsky S.V. (2005). Endothelial microparticles affect angiogenesis in vitro: Role of oxidative stress. Am. J. Physiol. Heart Circ. Physiol..

[16] Tang N., Sun B., Gupta A., Rempel H., Pulliam L. (2016). Monocyte exosomes induce adhesion molecules and cytokines via activation of NF-κB in endothelial cells. FASEB J..

[17] Słomka A., Urban S.K., Lukacs-Kornek V., Żekanowska E., Kornek M. (2018). Large extracellular vesicles: Have we found the holy grail of inflammation?. Front. Immunol..

[18] Kittivorapart J., Crew V.K., Wilson M.C., Heesom K.J., Siritanaratkul N., Toye A.M. (2018). Quantitative proteomics of plasma vesicles identify novel biomarkers for hemoglobin E/β-thalassemic patients. Blood Adv..

[19] Levin C., Koren A., Rebibo-Sabbah A., Koifman N., Brenner B., Aharon A. (2018). Extracellular Vesicle Characteristics in β-thalassemia as Potential Biomarkers for Spleen Functional Status and Ineffective Erythropoiesis. Front. Physiol..

[20] Mocan T., Simão A.L., Castro R.E., Rodrigues C.M.P., Słomka A., Wang B., Strassburg C., Wöhler A., Willms A.G., Kornek M. (2020). Liquid biopsies in hepatocellular carcinoma: Are we winning?. J. Clin. Med..

[21] Sundd P., Gladwin M.T., Novelli E.M. (2019). Pathophysiology of sickle cell disease. Annu. Rev. Pathol..

[22] Hoover R., Rubin R., Wise G., Warren R. (1979). Adhesion of normal and sickle erythrocytes to endothelial monolayer cultures. Blood.

[23] Hebbel R.P., Yamada O., Moldow C.F., Jacob H.S., White J.G., Eaton J.W. (1980). Abnormal adherence of sickle erythrocytes to cultured vascular endothelium: Possible mechanism for microvascular occlusion in sickle cell disease. J. Clin. Investig..

[24] Lapping-Carr G., Gemel J., Mao Y., Beyer E.C. (2020). Circulating extracellular vesicles and endothelial damage in sickle cell disease. Front. Physiol..

[25] Vats R., Brzoska T., Bennewitz M.F., Jimenez M.A., Pradhan-Sundd T., Tutuncuoglu E., Jonassaint J., Gutierrez E., Watkins S.C., Shiva S. (2020). Platelet Extracellular Vesicles Drive Inflammasome-IL-1β-Dependent Lung Injury in Sickle Cell Disease. Am. J. Respir. Crit. Care Med..

[26] Garnier Y., Ferdinand S., Garnier M., Cita K.-C., Hierso R., Claes A., Connes P., Hardy-Dessources M.-D., Lapouméroulie C., Lemonne N. (2020). Plasma microparticles of sickle patients during crisis or taking hydroxyurea modify endothelium inflammatory properties. Blood.

[27] Smith R.A., Mankelow T.J., Drizou D., Bullock T., Latham T., Trompeter S., Blair A., Anstee D.J. (2021). Large red cell-derived membrane particles are major contributors to hypercoagulability in sickle cell disease. Sci. Rep..

[28] Van Beers E.J., Schaap M.C.L., Berckmans R.J., Nieuwland R., Sturk A., van Doormaal F.F., Meijers J.C.M., Biemond B.J., CURAMA Study Group (2009). Circulating erythrocyte-derived microparticles are associated with coagulation activation in sickle cell disease. Haematologica.

[29] Shet A.S., Aras O., Gupta K., Hass M.J., Rausch D.J., Saba N., Koopmeiners L., Key N.S., Hebbel R.P. (2003). Sickle blood contains tissue factor-positive microparticles derived from endothelial cells and monocytes. Blood.

[30] Khalyfa A., Khalyfa A.A., Akbarpour M., Connes P., Romana M., Lapping-Carr G., Zhang C., Andrade J., Gozal D. (2016). Extracellular microvesicle microRNAs in children with sickle cell anaemia with divergent clinical phenotypes. Br. J. Haematol..

[31] Lapping-Carr G., Khalyfa A., Rangel S., Darlington W., Beyer E.C., Peddinti R., Cunningham J.M., Gozal D. (2017). Exosomes contribute to endothelial integrity and acute chest syndrome risk: Preliminary findings. Pediatr. Pulmonol..

[32] Lapping-Carr G., Gemel J., Mao Y., Sparks G., Harrington M., Peddinti R., Beyer E.C. (2020). Circulating extracellular vesicles from patients with acute chest syndrome disrupt adherens junctions between endothelial cells. Pediatr. Res..

[33] Gemel J., Mao Y., Lapping-Carr G., Beyer E.C. (2020). Gap Junctions between Endothelial Cells Are Disrupted by Circulating Extracellular Vesicles from Sickle Cell Patients with Acute Chest Syndrome. Int. J. Mol. Sci..

[34] Ludwig A.-K., Giebel B. (2012). Exosomes: Small vesicles participating in intercellular communication. Int. J. Biochem. Cell Biol..

[35] Momen-Heravi F., Balaj L., Alian S., Tigges J., Toxavidis V., Ericsson M., Distel R.J., Ivanov A.R., Skog J., Kuo W.P. (2012). Alternative methods for characterization of extracellular vesicles. Front. Physiol..

[36] Nader E., Garnier Y., Connes P., Romana M. (2021). Extracellular vesicles in sickle cell disease: Plasma concentration, blood cell types origin distribution and biological properties. Front. Med..

[37] Ghosh S., Tan F., Ofori-Acquah S.F. (2012). Spatiotemporal dysfunction of the vascular permeability barrier in transgenic mice with sickle cell disease. Anemia.

[38] Umapathy N.S., Gonzales J., Makala L.H., Xu H., Biddinger P., Pace B.S. (2017). Impaired pulmonary endothelial barrier function in sickle cell mice. Haematologica.

[39] Kato G.J., Steinberg M.H., Gladwin M.T. (2017). Intravascular hemolysis and the pathophysiology of sickle cell disease. J. Clin. Investig..

[40] Singla S., Sysol J.R., Dille B., Jones N., Chen J., Machado R.F. (2017). Hemin causes lung microvascular endothelial barrier dysfunction by necroptotic cell death. Am. J. Respir. Cell Mol. Biol..

[41] Santaterra V.A.G., Fiusa M.M.L., Hounkpe B.W., Chenou F., Tonasse W.V., da Costa L.N.G., Garcia-Weber D., de Farias Domingos I., de Lima F., Borba-Junior I.T. (2020). Endothelial barrier integrity is disrupted in vitro by heme and by serum from sickle cell disease patients. Front. Immunol..

[42] Meegan J.E., Bastarache J.A., Ware L.B. (2021). Toxic effects of cell-free hemoglobin on the microvascular endothelium: Implications for pulmonary and nonpulmonary organ dysfunction. Am. J. Physiol. Lung Cell Mol. Physiol..

