# Supplementary material for: Circulating Small Extracellular Vesicles May Contribute to Vaso-Occlusive Crises in Sickle Cell Disease
Source: J Clin Med. 2022 Feb 3;11(3):816. doi: 10.3390/jcm11030816 (PMC8836895; doi:10.3390/jcm11030816)
Supplement: Supplementary file 1 [file jcm-11-00816-s001.zip › jcm-1515454-supplementary.pdf]

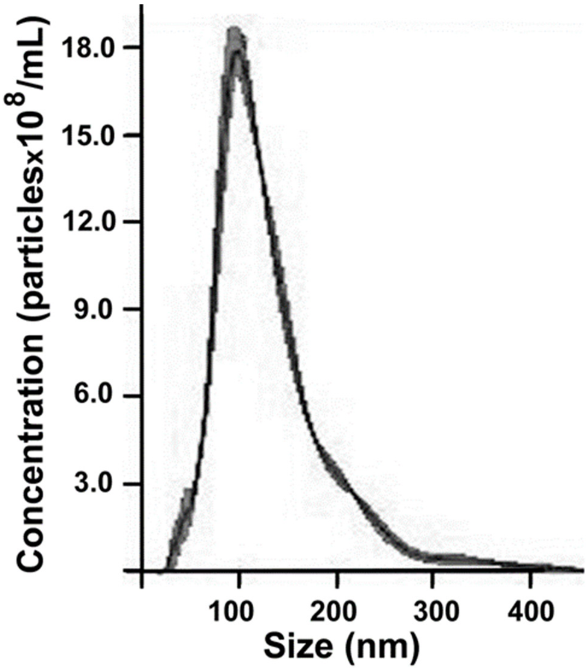

Figure S1. Representative Nanoparticle Tracking Analysis profile of EVs isolated from platelet-free plasma of a SCD patient. The mode peak size of the EVs is 98 nm.

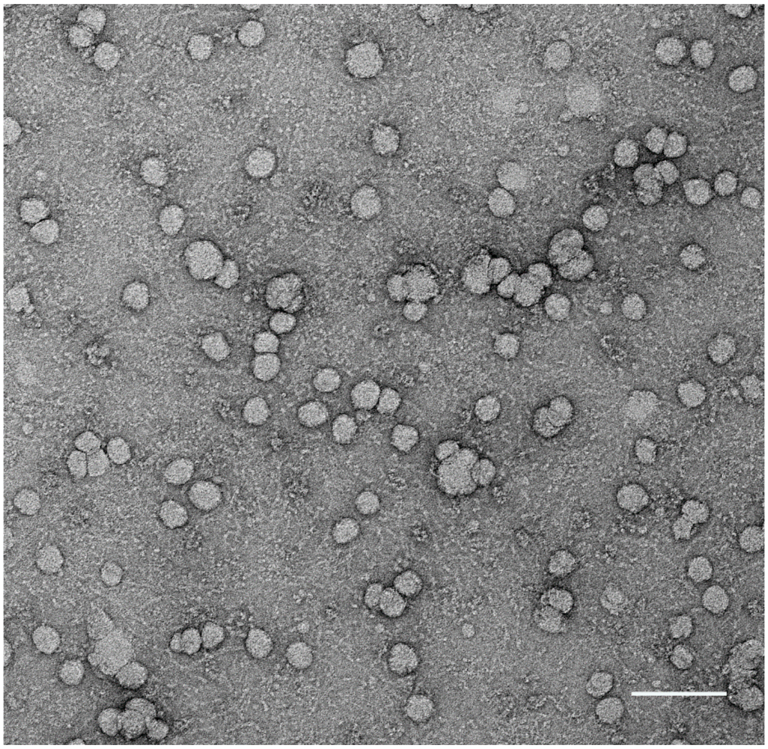

Figure S2. Transmission electron micrograph illustrates the appearance of EVs isolated by size exclusion chromatography from the platelet-free plasma of a patient with SCD after negative staining. Scale bar is 100 nm.

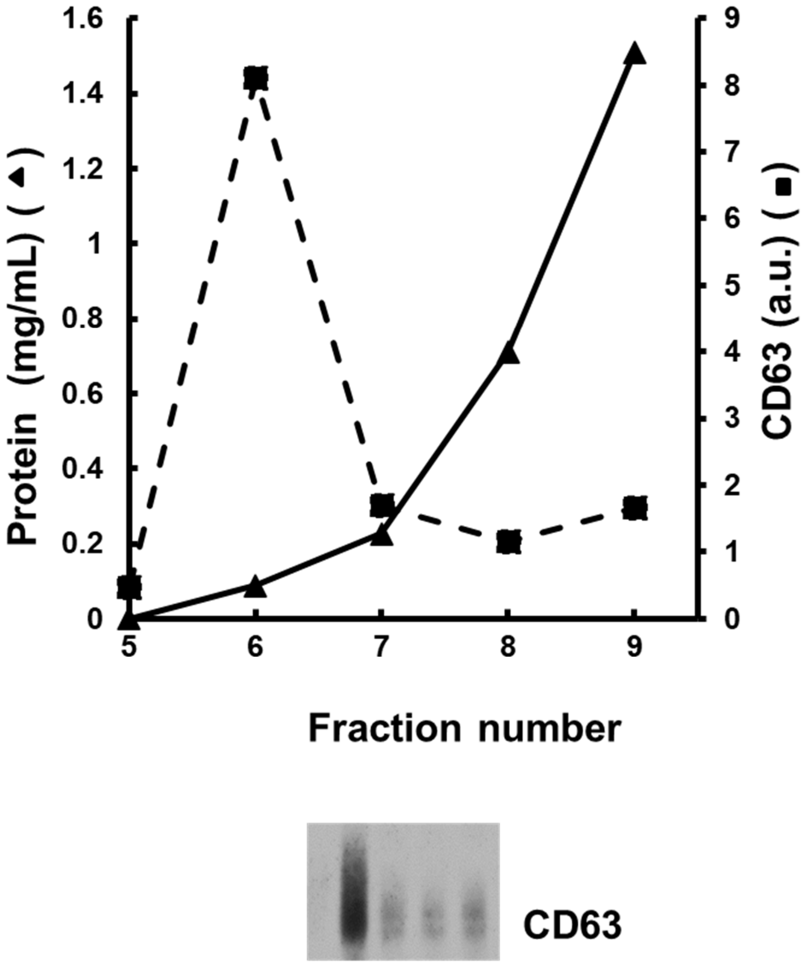

Figure S3. Properties of EVs isolated by size exclusion chromatography. 200 µl of platelet-free plasma from a subject with SCD. was applied to a size exclusion column. The eluted fractions were tested for total protein (by Coomassie blue dye binding) and for CD63 and flotillin (by immunoblotting). Immunoreactive CD63 bands were quantified by densitometry. The graph (A) shows the elution profiles for total protein and CD63. Panel B shows the immunoblot detection of CD63 and flotillin in various fractions. The peak of CD63 was found in fraction 6, while the bulk of the protein was present in much later fractions. Flotillin was detectable starting from fraction 6, but substantial amounts were present in subsequent fractions, consistent with pat observations that it is present in exosomes and in non-exosomal materials.

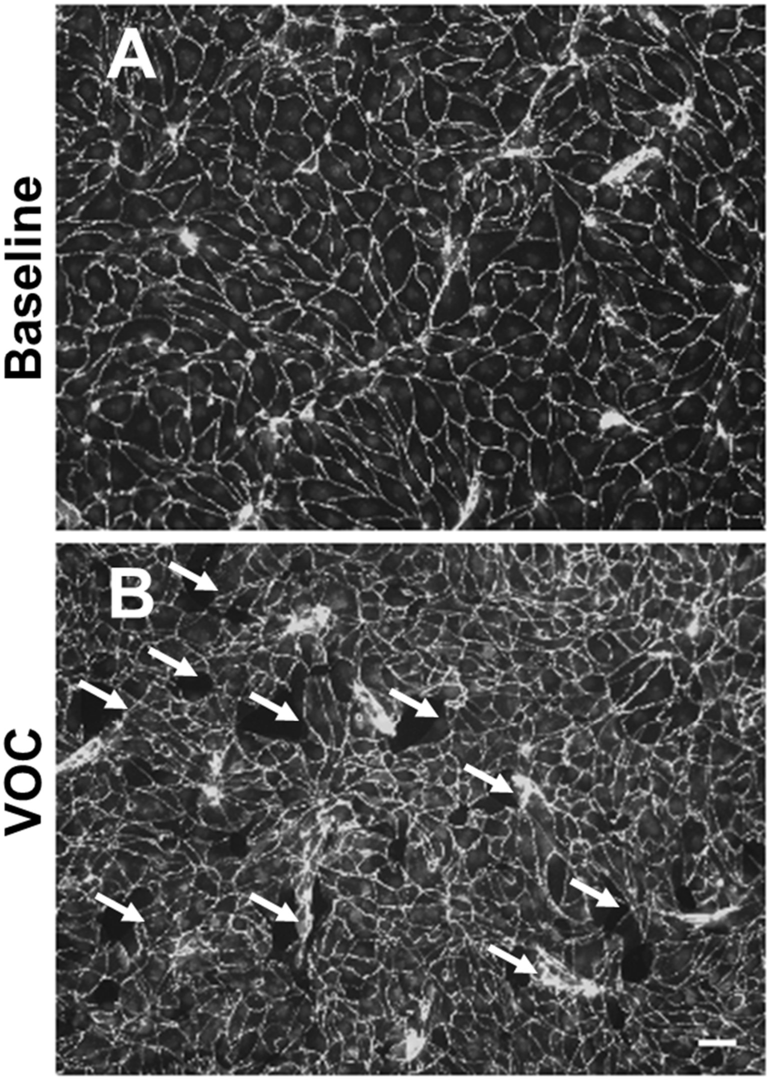

Figure S4. Representative low magnification photomicrographs of endothelial monolayer integrity and its disruption by EVs. Photomicrographs were obtained from cells 48 h after treatment with EVs from a subject with SCD at baseline (A) or with EVs from the same subject at the beginning of an episode of VOC (B). VE-cadherin was detected by immunofluorescence. In the examples shown, the baseline sample showed no disruption of the monolayer; for the VOC sample, monolayer disruption was 3.9%. The white arrows indicate cell-free spaces that have opened between cells. Scale bar represents 50 μm.

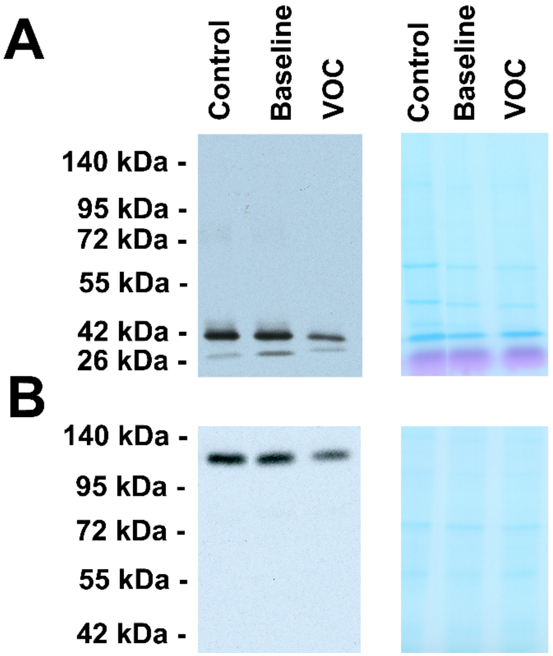

Figure S5. Levels of Cx43 and VE-cadherin are decreased in endothelial.**cells treated with EVs obtained during a VOC episode.** EVs were isolated by precipitation method. Endothelial cells were grown to confluence and then treated with EVs from a control subject, EVs from a subject with SCD (obtained at baseline), or EVs from the same subject (obtained during a VOC episode). After 48 hours, cell lysates were prepared, and proteins were resolved by SDS-PAGE. Cx43 or VE-cadherin were detected by immunoblotting. Left panels show immunoblot detection of Cx43 (A) and VE-cadherin (B). Right panels show MEM Code Reversible Protein Staining of the corresponding PVDF membranes. In these examples, densitometry showed that levels of Cx43 and VE-cadherin differed by <5% between baseline and controls. However, the abundance of Cx43 was decreased by 49%, and the level of VE-cadherin was decreased by 36% between baseline and VOC.
